# A comparative analysis of ACE Inhibitors and ARBs as cancer risk factors

**DOI:** 10.1371/journal.pone.0354248

**Published:** 2026-07-23

**Authors:** Belle Tamir Brahms, Shani Afek, Michael Hopp, Martine Granek-Catarivas, Yair Levy, Tzipi Hornik-Lurie

**Affiliations:** 1 Department of Family Medicine, Clalit Health Services, Sharon-Shomron, Israel; 2 Dina Recanati School of Medicine, Reichman University, Herzliya, Israel; 3 Omnistat & Tel Aviv University, Tel Aviv, Israel; 4 Gray Faculty of Medical & Health Sciences, Tel Aviv University, Tel Aviv, Israel; 5 Internal Medicine Department E, Meir Medical Center Research Institute, Clalit Health Services, Kfar Saba, Israel; University of British Columbia, CANADA

## Abstract

**Importance:**

Angiotensin-converting enzyme inhibitors (ACEIs) and angiotensin receptor blockers (ARBs) are widely prescribed antihypertensive agents. Although their cardiovascular and renal benefits are well established, uncertainty remains regarding their long-term associations with cancer risk.

**Objective:**

To compare cancer incidence among patients treated exclusively with ACEIs or ARBs in a large population-based cohort with long-term follow-up.

**Design, setting, and participants:**

Retrospective cohort study using electronic health records from Clalit Health Services, Israel’s largest healthcare organization. The source population included hypertensive adults aged 40 years or older who were free of cancer on January 1, 2000 (n = 1,048,575). After applying eligibility criteria and excluding patients who switched between treatment groups or were non-compliant with therapy, the final analytic cohort included 346,405 exclusive ACEI users and 77,018 exclusive ARB users.

**Exposure:**

Exclusive treatment with either ACEIs or ARBs. Cancer incidence analyses were conducted during an active follow-up period from 2002 through 2024, allowing for a two-year exposure window.

**Main outcomes and measures:**

The primary outcome was diagnosis of any cancer. Secondary outcomes included the ten most prevalent cancer types diagnosed during follow-up. Cancer incidence rates, odds ratios (ORs), age at diagnosis, and time from treatment initiation to cancer diagnosis were compared between treatment groups after adjustment for age, sex, and exposure duration.

**Results:**

During follow-up, overall cancer incidence differed between treatment groups. ACEI therapy was associated with lower overall odds of cancer than ARB therapy. Associations varied across specific cancer types, with lower odds observed among ACEI users for several common malignancies, whereas no meaningful difference was observed for leukemia. Differences in age at diagnosis and time to diagnosis were also observed between treatment groups.

**Conclusions and relevance:**

In this large population-based cohort, long-term cancer risk differed between patients treated exclusively with ACEIs and those treated exclusively with ARBs. While the observational design precludes causal inference, the findings contribute to the ongoing evaluation of the long-term oncologic safety profiles of RAAS-modulating therapies. Further prospective and mechanistic studies are needed to clarify the biological basis and clinical significance of these associations.

## Introduction

Angiotensin-converting enzyme inhibitors (ACEIs) and angiotensin receptor blockers (ARBs) are cornerstone therapies in the management of cardiovascular and renal diseases [1–3]. Although both drug classes act on the renin–angiotensin–aldosterone system (RAAS), they do so through distinct pharmacological mechanisms [4,5]. While their cardiovascular and renal benefits are well established, concerns regarding their potential association with cancer risk remain unresolved [6,7].

Experimental evidence suggests that angiotensin-II receptors may play a role in angiogenesis, cellular proliferation, inflammation, and tumor progression, providing plausible biological mechanisms through which RAAS-modulating therapies could influence cancer development [8]. Initial concerns regarding a possible association between ARB use and cancer risk emerged following the CHARM trial and were subsequently explored in multiple observational studies and meta-analyses; however, findings have remained inconsistent [9–16].

Further complicating the safety profile of ARBs, probable human carcinogens including N-nitrosodimethylamine (NDMA), N-nitrosodiethylamine (NDEA), and azido-related impurities were identified in several ARB formulations beginning in 2018, leading to widespread product recalls and increased regulatory scrutiny. These manufacturing-related impurities, detected in products containing valsartan, losartan, and irbesartan, have raised additional concerns regarding the long-term safety of certain ARB formulations [17–21].

Evidence regarding ACEIs has also been mixed. While several studies have not demonstrated a clear association between ACEI use and overall cancer risk [22], more recent investigations have reported an increased risk of lung cancer among ACEI users compared with ARB users, particularly in Asian populations [23–25]. Conversely, other analyses have suggested that prolonged exposure to ARBs may be associated with increased cancer risk [26]. Additional studies have reported conflicting findings regarding the relationship between ACEI use and specific cancer types [14,27,28].

Despite extensive investigation, important gaps in the literature remain. Many previous studies focused on specific malignancies, included relatively limited follow-up periods, or evaluated heterogeneous patient populations with varying patterns of exposure to ACEIs and ARBs. Furthermore, few studies have examined long-term cancer outcomes among patients treated exclusively with one drug class while accounting for duration of exposure. Consequently, the comparative cancer risk associated with prolonged exclusive use of ACEIs versus ARBs remains incompletely understood.

Therefore, we conducted a large population-based cohort study using electronic health records from Clalit Health Services, Israel’s largest healthcare organization, with up to 24 years of follow-up. The aim of this study was to compare cancer incidence among patients treated exclusively with ACEIs or ARBs and to evaluate whether long-term cancer risk differs between these two commonly prescribed RAAS-modulating therapies.

## Study population

This study compared cancer incidence among patients treated exclusively with angiotensin-converting enzyme inhibitors (ACEIs) or angiotensin receptor blockers (ARBs). It forms part of a broader investigation examining the relationship between antihypertensive treatment, and cancer incidence.

Data were obtained from the electronic health records of Clalit Health Services, Israel’s largest healthcare organization, serving approximately 4.8 million members. The database includes longitudinal information on demographic characteristics, clinical diagnoses, medication dispensing records, laboratory results, procedures, and mortality.

The study protocol was approved by the relevant institutional ethics committee in accordance with the Declaration of Helsinki (approval number:0130-23-COM2) The data analyzed in this study were obtained from Clalit Health Services and contain sensitive patient information.

The dataset used in this study was extracted by the Research Unit of Meir Medical Center, Sharon–Shomron District.

The source population consisted of all patients aged 40 years or older with no prior cancer diagnosis as of January 1, 2000 (n = 1,048,575). Participants were followed retrospectively through December 31, 2024. Demographic characteristics, smoking status, socioeconomic status, body mass index (BMI), comorbidity burden, medication use, mortality, and incident cancer diagnoses were extracted from the database.

**Fig 1:**
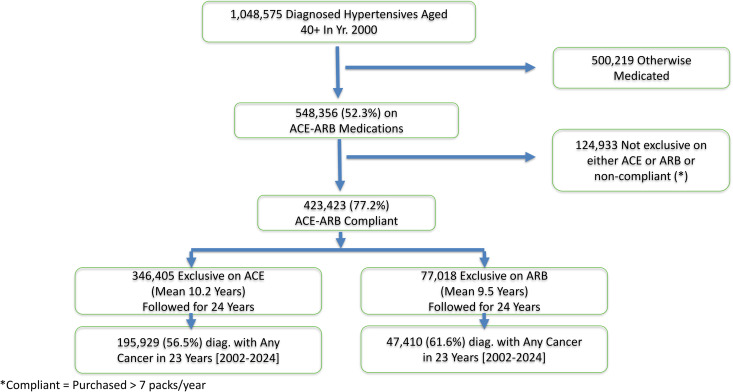
Study population; Sample Selection Process.

For the present analysis, individuals who received exclusively ACEIs or exclusively ARBs during follow-up were identified. Patients who had received either medication class before January 1, 2000, those who switched between ACEIs and ARBs during follow-up, and those classified as non-compliant with treatment were excluded. Compliance was defined as the purchase of more than seven medication packs per year. The sample selection process is illustrated in Fig 1.

To ensure a minimum exposure period and reduce the potential for reverse-causation bias, cancer incidence analyses were initiated on January 1, 2002, allowing for a two-year exposure window after cohort entry. Participants were subsequently followed for incident cancer outcomes through December 31, 2024, providing up to 23 years of active cancer follow-up.

The final analytic cohort included 346,405 patients treated exclusively with ACEIs and 77,018 treated exclusively with ARBs. Comparative analyses were conducted to evaluate the association between treatment group and the incidence of the ten most prevalent cancer types diagnosed during follow-up. Cancer incidence rates were calculated annually and averaged across the follow-up period to account for differences in exposure duration and population size over time. Age-, sex-, and exposure-adjusted odds ratios (ORs), 95% confidence intervals (CIs), and corresponding p-values were calculated to compare cancer risk between treatment groups.

### Analysis

Data were extracted using MDClone (https://www.mdclone.com), exported to Microsoft Excel, and analyzed using IBM SPSS Statistics version 29. Cancer incidence was evaluated separately for the ten most prevalent cancer types identified during the follow-up period. Analyses were restricted to patients treated exclusively with either ACEIs or ARBs.

Researchers employed by Clalit Health Services may access the data following approval by the relevant Clalit Helsinki Committee and all applicable institutional procedures. External researchers may request access through collaboration with a Clalit-affiliated investigator and subject to the same ethical and institutional approval requirements.To account for differences in age, sex, and duration of medication exposure, annual cancer incidence rates were calculated separately for each treatment group and averaged across the follow-up period. Absolute incidence rates and age-, sex-, and exposure-adjusted odds ratios (ORs) with corresponding 95% confidence intervals (CIs) and p-values were calculated to compare cancer risk between ACEI and ARB users. Summary results are presented in Tables 2–4.

### General findings

Among 1,048,575 hypertensive adults aged 40 years or older and free of cancer at baseline, 548,356 (52.3%) received ACEIs or ARBs during the study period (Fig 1). After excluding patients who switched between medication classes or were classified as non-compliant, the final analytic cohort included 346,405 individuals treated exclusively with ACEIs and 77,018 treated exclusively with ARBs.

Mean duration of treatment was 10.2 years (SD 6.12) among ACEI users and 9.5 years (SD 5.55) among ARB users. Baseline demographic and clinical characteristics of the study population are presented in Table 1.

**Table 1:** Study population by ACE/ARB Status, Social, Physiol & Medical Particulars.

		ACE OR ARB						P-Val
		ACE		ARB		Total		
		N	Col %	N	Col %	N	Col %	
**TOTAL**		**346405**	**100.0%**	**77018**	**100.0%**	**423423**	**100.0%**	
Sex	Female	175638	50.7%	48743	63.3%	224381	53.0%	<.001^*^
	Male	170767	49.3%	28275	36.7%	199042	47.0%	
Socioeconomic Score	Very Low	12234	3.5%	1489	1.9%	13723	3.2%	<.001^*^
	Low	91004	26.3%	14667	19.0%	105671	25.0%	
	Medium	133764	38.6%	29645	38.5%	163409	38.6%	
	HIGH	81205	23.4%	22195	28.8%	103400	24.4%	
	Very High	28198	8.1%	9022	11.7%	37220	8.8%	
Age In Year 2000	Mean (SD)	61.0	[13.05]	56.5	[10.80]	60.2	[12.78]	<.001*
Age in 2024	Mean (SD)	75.3	[7.76]	75.9	[7.65]	75.5	[7.74]	<.001*
Dead Or Alive in 2024	Dead	192313	55.5%	25117	32.6%	217430	51.4%	<.001^*^
	Alive	154092	44.5%	51901	67.4%	205993	48.6%	
Age at Death	Mean (SD)	82.4	[9.80]	83.3	[9.08]	82.5	9.7	<.001*
Smoking Status	Smokes	92957	26.8%	19582	25.4%	112539	26.6%	<.001^*^
	Does Not Smoke	253448	73.2%	57436	74.6%	310884	73.4%	
BMI Before 2012	Mean (SD)	28.5	[5.16]	28.9	[5.06]	28.6	[5.15]	<.001*
BMI After 2012	Mean (SD)	27.9	[5.19]	28.7	[5.22]	28.1	[5.21]	<.001*
Charlson comorbidy 2020−2012	Mean (SD)	1.4	[1.28]	1.3	[1.29]	1.3	[1.29]	<.001*
Charlson comorbidy 2012–2024	Mean (SD)	2.8	[1.70]	2.8	[1.71]	2.8	[1.70]	0.927
Years on ACE or ARB	Mean (SD)	10.2	[6.12]	9.5	[5.55]	10.1	[6.03]	<.001*
Years with Hypertension	Mean (SD)	20.0	[3.69]	19.7	[4.32]	20.0	[3.80]	0.000
Years with Atrial Fibrilation	Mean (SD)	19.7	[5.43]	19.6	[6.00]	19.7	[5.52]	0.021
Cancers Developed 2002–2024	Any Cancer	195929	56.5%	47410	61.6%	243339	57.5%	<.001^*^
	No Cancer	150476	43.5%	29608	38.4%	180084	42.5%	

During the active cancer follow-up, 56.5% of ACEI users and 61.6% of ARB users were diagnosed with some type of cancer.

Group differences in baseline characteristics were assessed using chi-square tests for categorical variables and independent-samples t-tests for continuous variables.

### Cancer incidence

During the follow-up period, a total of 665,605 cancer diagnoses were recorded in the study population, excluding recurrences and including multiple primary cancer diagnoses occurring in the same individual. The ten most prevalent cancer types identified during follow-up are presented in Table 2.

**Table 2:** 23 year incidence of the most prevalent cancer diagnoses among ACE/ARB users, presented in descending order.

	2002-2011 Period (*)	2012-2024 Period (**)	Total Cases 2002–2024	Mean Incidence/000/y	Mean Age at Diagnosis	on ACE Medication		on ARB Medication	
	2002-2011 Cases	2012-2024 Cases				Cases	Mean Incidence/000/y	Cases	Mean Incidence/000/y
Breast Cancer (+)	15524	23938	39462	3.78	69.1	16279	5.27	5273	9.66
Prostate Cancer (++)	13603	16315	29918	3.29	70.5	14969	14.65	2888	10.87
Lung Cancer	10468	16734	27202	1.35	70.6	12080	2.14	2375	1.04
Melanoma	6628	4701	11329	0.65	69.7	4212	0.88	1267	0.44
Bladder Cancer	6116	6103	12219	0.64	67.1	5639	1.15	1026	0.88
Leukemia (Chronic & Acute)	3944	1260	5204	0.45	68.1	3176	0.50	681	0.77
Pancreatic Cancer	2068	2897	4965	0.25	70.6	1875	0.41	366	0.20
Kidney Cancer	2473	3207	5680	0.16	70.9	2445	0.51	608	0.47
Colorectal Cancer	2141	2307	4448	0.15	71.1	1624	0.35	411	0.33
Non-Hodgkins Lymphoma	1501	1385	2886	0.24	70.3	7766	0.21	1962	0.34
Other & unspecified Cancers	129074	132072	261146	0.96	70.5	125729	26.40	30565	41.65
**Initial Exposed Population**	**1048575**	**869667**				**346405**		**77018**	
**All Cause Deaths**	178908	245773				**192313**		**25117**	
**Mean extant population**	914452	704256				**190520**		**61735**	

(*) First 10 Year Followup Period.

(**) Second 13 Year Followup Period.

(+) Women Breast Cancer Only.

(++) Men Only.

Breast cancer in women, prostate cancer in men, and lung cancer in both sexes accounted for approximately two-thirds of all cancer diagnoses among the most common malignancies observed. The overall mean age at cancer diagnosis was 70.4 years (SD 10.45).

To account for differences in treatment duration and changes in the population at risk over time, annual incidence rates were calculated separately for each treatment group and averaged across the 23-year follow-up period.

### Association between ACEI/ARB therapy and cancer incidence

Adjusted odds ratios comparing ACEI and ARB users for the ten most prevalent cancer types are presented in Table 3. Overall, ACEI therapy was associated with lower odds of developing cancer than ARB therapy (OR 0.851, 95% CI 0.838–0.865).

**Table 3:** ACE to ARB Odds Ratio for the 10 Most Prevalent Invasive Cancers.

	O.R.	95% CI			P. VAL
Breast	0.830	0.803	–	0.858	0.000
Prostate	0.841	0.807	–	0.878	0.000
Lung	1.136	1.086	–	1.188	0.000
N.H. Lymphoma	0.877	0.834	–	0.922	0.000
Bladder	1.226	1.146	–	1.311	0.000
Melanoma	0.736	0.691	–	0.784	0.000
Leukemia	1.037	0.955	–	1.127	0.200
Kidney	0.893	0.817	–	0.977	0.007
Pancreas	1.140	1.019	–	1.275	0.012
Colorectal	0.878	0.788	–	0.979	0.010
**All Cancers**	**0.851**	**0.838**	–	**0.865**	**0.000**

The direction and magnitude of associations varied across cancer types. Lower odds among ACEI users were observed for breast, prostate, melanoma, non-Hodgkin lymphoma, kidney, and colorectal cancers. In contrast, higher odds were observed for lung, bladder, and pancreatic cancers. No statistically significant difference was observed for leukemia.

These findings indicate that the relationship between RAAS-modulating therapies and cancer risk may differ according to cancer type.

### Development of cancer risk over time and ACEI/ARB exposure

**Fig 2:**
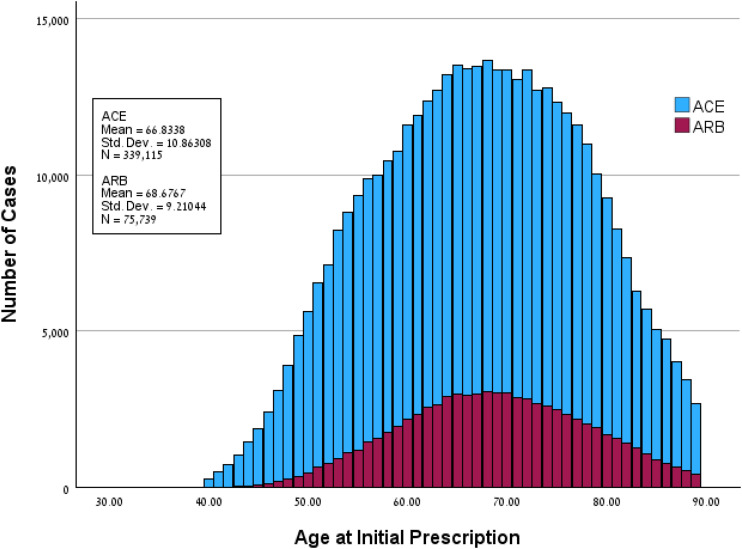
Age distributing of ACE & ARB initial prescription.

Although the analysis was restricted to patients treated exclusively with either ACEIs or ARBs, a modest difference in age at treatment initiation was observed between groups. Mean age at first prescription was 66.8 years (SD 10.8) among ACEI users and 68.7 years (SD 9.2) among ARB users (p < 0.001) (Fig 2 and Table 4).

**Table 4:** Summarizes the time from treatment initiation to cancer diagnosis and age at diagnosis for the three most prevalent cancer types.

Cancer		Years	SD	P.Val	Age	SD	P.Val	N_Treated
Breast	ACE	10.84	[6.59]	P > .000	71.08	[11.31]	P > .001	175638
	ARB	8.66	[5.51]		70.10	[11.13]		48743
Prostate	ACE	11.11	[6.55]	P > .000	72.63	[8.48]	P > .001	170767
	ARB	8.28	[5.44]		71.07	[8.48]		28275
Lung	ACE	9.8	[6.28]	P > .000	73.37	[9.33]	NS	322043
	ARB	7.1	[5.11]		73.54	[9.37]		99412

Across the most prevalent cancer types, ACEI users generally experienced longer intervals between treatment initiation and cancer diagnosis than ARB users. Similarly, the mean age at cancer diagnosis was higher among ACEI users for most cancer types, although differences were not statistically significant for lung cancer and leukemia.

**Fig 3:**
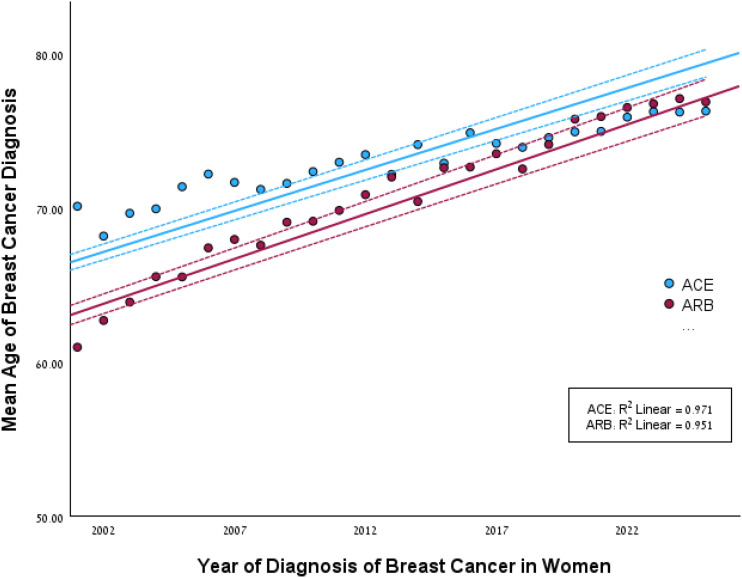
23 year tren of mean age breast cancer diagnosis in a stationary sample, by ACE & ARB.

Fig 3 illustrates temporal trends in mean age at breast cancer diagnosis among ACEI and ARB users during follow-up. Mean age at diagnosis increased over time in both groups, while differences between treatment groups became less pronounced in later years of follow-up.

## Discussion

Previous large-scale studies comparing the oncologic safety profiles of angiotensin-converting enzyme inhibitors (ACEIs) and angiotensin receptor blockers (ARBs) have reported inconsistent findings, as outlined in the Introduction. To further investigate this question, we conducted a large population-based cohort study evaluating cancer incidence among patients treated exclusively with ACEIs or ARBs over an extended follow-up period.

Overall, ACEI therapy was associated with lower odds of cancer compared with ARB therapy. However, the magnitude and direction of the associations varied across cancer types. Lower odds among ACEI users were observed for several common malignancies, including breast, prostate, melanoma, non-Hodgkin lymphoma, kidney, and colorectal cancers, whereas higher odds were observed for lung, bladder, and pancreatic cancers. No meaningful difference was observed for leukemia. These findings suggest that the relationship between RAAS-modulating therapies and cancer risk may differ according to cancer type.

The prolonged follow-up period enabled assessment of long-term patterns of cancer occurrence. Although patients treated with ACEIs were, on average, younger at treatment initiation than patients treated with ARBs, they generally experienced longer intervals between treatment initiation and cancer diagnosis. Furthermore, for most cancer types, the mean age at diagnosis was higher among ACEI users. Together, these findings suggest that differences in age at treatment initiation alone are unlikely to fully explain the observed associations. Nevertheless, residual confounding cannot be excluded.

An important observation is that several of the cancer types demonstrating differences between treatment groups are malignancies for which established screening strategies are available. While the present study was not designed to evaluate screening effectiveness, these findings may have implications for future research examining whether cancer surveillance strategies should be considered when assessing the long-term safety profiles of antihypertensive therapies.

This study has several notable strengths. It was based on a large real-world cohort derived from a comprehensive healthcare database and included more than 423,000 patients treated exclusively with ACEIs or ARBs. The long follow-up period of up to 23 years allowed assessment of long-term cancer outcomes that may not be detectable in shorter studies. In addition, the analysis evaluated multiple cancer types within the same population, providing a broad comparison of the oncologic profiles of the two medication classes. Adjustment for age, sex, and duration of exposure further strengthened the analysis.

Several limitations should also be considered. First, as an observational retrospective cohort study, the analysis cannot establish causality. Second, despite adjustment for important demographic and exposure-related factors, residual confounding remains possible. Treatment allocation was not random, and prescribing patterns may have differed according to clinical characteristics that were not fully captured in the available data. Third, the study population was derived from a single national healthcare organization and may not be fully representative of other healthcare systems or populations. Finally, although efforts were made to account for differences in exposure duration and age, the potential influence of unmeasured clinical and behavioral factors cannot be completely excluded.

Taken together, these findings suggest that long-term cancer risk may differ between ACEIs and ARBs and that these differences vary across specific malignancies. Further prospective studies and mechanistic investigations are needed to clarify the biological pathways underlying these associations and to determine whether the observed relationships reflect causal effects, residual confounding, or a combination of both.

## Conclusions

In this large population-based cohort with up to 23 years of follow-up, ACEI therapy was associated with lower overall odds of cancer compared with ARB therapy. However, the magnitude and direction of the associations varied across specific cancer types, suggesting that the relationship between RAAS-modulating therapies and cancer risk may be complex and disease-specific.

Given the observational nature of the study, causal inferences cannot be drawn. Nevertheless, the findings contribute to the ongoing discussion regarding the long-term oncologic safety profiles of ACEIs and ARBs and highlight the importance of considering potential differences between these commonly prescribed drug classes.

Further prospective studies and mechanistic investigations are needed to clarify the biological pathways underlying these associations and to determine whether the observed differences reflect causal effects, residual confounding, or other factors. Improved understanding of these relationships may support more informed clinical decision-making for patients requiring long-term RAAS-modulating therapy.

### Clinical implications

Given the widespread use of ACEIs and ARBs in the management of hypertension and other chronic conditions, even modest differences in long-term cancer risk may have important public health implications. In this large population-based cohort, overall cancer risk differed between patients treated exclusively with ACEIs and those treated exclusively with ARBs, although the magnitude and direction of the associations varied across specific cancer types.

The findings do not support changes in current prescribing recommendations but contribute additional evidence to the ongoing evaluation of the long-term safety profiles of RAAS-modulating therapies. When selecting antihypertensive treatment, clinicians should continue to consider established cardiovascular and renal benefits while remaining attentive to emerging evidence regarding potential long-term outcomes.

Several of the cancer types examined in this study are associated with established screening programs. Although the present study was not designed to evaluate screening effectiveness, the observed differences in cancer incidence may warrant further investigation in future studies examining the interaction between antihypertensive treatment, cancer risk, and opportunities for early detection.

These findings reinforce the importance of continued pharmacovigilance and highlight the need for prospective studies to clarify the clinical significance of the observed associations.
